# Supplementary material for: Strand-resolved mutagenicity of DNA damage and repair
Source: Nature. 2024 Jun 12;630(8017):744–51. doi: 10.1038/s41586-024-07490-1 (PMC11186772; doi:10.1038/s41586-024-07490-1)
Supplement: Supplementary file 1 — Reporting Summary [file 41586_2024_7490_MOESM1_ESM.pdf]

nature portfolio

Corresponding author(s): Martin Taylor
Duncan Odom
Sarah Aitken

Last updated by author(s): Mar 28, 2024

Reporting Summary

Nature Portfolio wishes to improve the reproducibility of the work that we publish. This form provides structure for consistency and transparency in reporting. For further information on Nature Portfolio policies, see our Editorial Policies and the Editorial Policy Checklist.

Statistics

For all statistical analyses, confirm that the following items are present in the figure legend, table legend, main text, or Methods section.

n/a	Confirmed	
☐	☒	The exact sample size (*n*) for each experimental group/condition, given as a discrete number and unit of measurement
☐	☒	A statement on whether measurements were taken from distinct samples or whether the same sample was measured repeatedly
☐	☒	The statistical test(s) used AND whether they are one- or two-sided<br>*Only common tests should be described solely by name; describe more complex techniques in the Methods section.*
☐	☒	A description of all covariates tested
☐	☒	A description of any assumptions or corrections, such as tests of normality and adjustment for multiple comparisons
☐	☒	A full description of the statistical parameters including central tendency (e.g. means) or other basic estimates (e.g. regression coefficient) AND variation (e.g. standard deviation) or associated estimates of uncertainty (e.g. confidence intervals)
☐	☒	For null hypothesis testing, the test statistic (e.g. *F*, *t*, *r*) with confidence intervals, effect sizes, degrees of freedom and *P* value noted<br>*Give P values as exact values whenever suitable.*
☒	☐	For Bayesian analysis, information on the choice of priors and Markov chain Monte Carlo settings
☐	☒	For hierarchical and complex designs, identification of the appropriate level for tests and full reporting of outcomes
☐	☒	Estimates of effect sizes (e.g. Cohen's *d*, Pearson's *r*), indicating how they were calculated

Our web collection on statistics for biologists contains articles on many of the points above.

Software and code

Policy information about availability of computer code

Data collection: Illumina Software Control (ICS) v.3.3.76

Data analysis:
Software and versions used in the analysis (fully detailed in Supplemental Table 3):
bwa mem 0.7.17 Li and Durbin, 2009
Bedtools intersect 2.29.2 Quinlan and Hall, 2010
cutadapt 2.6 Martin 2011
FIMO (MEME suite) 5.0.5 Grant et al, 2011
MACS2 2.1.2 Zhang et al., 2008
picard 2.23.8 Broad Institute, 2019
Python 3.9.7
QuPath 0.2.2 Bankhead et al., 2017
R 3.6.3 and 4.0.5 R Core Team, 2017
r-data.table 1.12.8 R Core Team, 2017
r-GreyListChIP unversionned Brown, 2021
r-segmented 1.3-3 Muggeo, 2003
r-sigFit 2.0 Gori and Baez-Ortega, 2018
Samtools 1.7 and 1.9 Li et al., 2009
SciKit-Learn 1.0.2 Pedregosa et al., 2011
SciPy 1.7.1 Virtanen et al., 2020
snakemake 6.1.1 Mölder et al., 2021

StarDist unversionned Schmidt et al., 2018<br>UCSC liftOver 377 Hinrichs et al., 2006

For manuscripts utilizing custom algorithms or software that are central to the research but not yet described in published literature, software must be made available to editors and reviewers. We strongly encourage code deposition in a community repository (e.g. GitHub). See the Nature Portfolio guidelines for submitting code & software for further information.

Data

Policy information about availability of data

All manuscripts must include a data availability statement. This statement should provide the following information, where applicable:

- Accession codes, unique identifiers, or web links for publicly available datasets
- A description of any restrictions on data availability
- For clinical datasets or third party data, please ensure that the statement adheres to our policy

Raw data files for all new datasets are available from Array Express (AE) and the European Nucleotide Archive (ENA) at EMBL-EBI. E/L Repli-seq accession numbers ENA: PRJEB67994, PRJEB72349; ATAC-seq accession number AE: E-MTAB-11780; ChIP-seq accession number AE: E-MTAB-11959.

Research involving human participants, their data, or biological material

Policy information about studies with human participants or human data. See also policy information about sex, gender (identity/presentation), and sexual orientation and race, ethnicity and racism.

Reporting on sex and gender	Not applicable
Reporting on race, ethnicity, or other socially relevant groupings	Not applicable
Population characteristics	Not applicable
Recruitment	Not applicable
Ethics oversight	Not applicable

Note that full information on the approval of the study protocol must also be provided in the manuscript.

Field-specific reporting

Please select the one below that is the best fit for your research. If you are not sure, read the appropriate sections before making your selection.

☒ Life sciences ☐ Behavioural & social sciences ☐ Ecological, evolutionary & environmental sciences

For a reference copy of the document with all sections, see nature.com/documents/nr-reporting-summary-flat.pdf

Life sciences study design

All studies must disclose on these points even when the disclosure is negative.

Sample size	Primary data for the study was previously generated and reported whole genome sequence for mouse DEN induced tumours, the full dataset comprised 371 biological replicates (Aitken et al, Nature 2020). ATAC-seq was performed using 3 biological replicates. ChIP-seq was performed using 5 biological replicates.
Data exclusions	Except where otherwise stated (within the final results section), analyses were confined to n=237, clonally distinct DEN induced tumours that met the combined criteria of: (i) not labelled as symmetric, (ii) tumour cellularity >50%, and (iii) >80% of substitution mutations attributed to the DEN1 signature by sigFit (v.2.0). Data exclusions were pre-defined based in previously reported frequency distributions of mutation spectra and variant allele frequency in the primary data (Aitken et al., Nature 2020).
Replication	Replication through the orthogonal validation of conclusions. Mutation rates and mutation spectra (successful). Mutation rates and multiallelic variation (successful). Symmetric tumour validation of NER-TRIM (successful). DNA accessibility findings replicated at nucleosomes, CTCF binding sites and transcription factor binding sites. Repli-Seq analysis was replicated with two independent cell lines.
Randomization	This study did not involve a randomised case:control study design. Randomisation was used to permute genes and tumour classifications for permutation based analysis (using the R sample function without replacement). Randomisation was used for bootstrap analyses (R sample function with replacement). Analysis code provided uses a defined seed for random number generation for reproducibility but equivalent results in each case were obtained using pseudo-randomly generated seeds.
Blinding	Researchers were blinded during histological scoring of tumours in prior work that generated the primary data for this study (Aitken et al., Nature 2020). Blinding was not relevant for genomic analyses as all processing was automated and all samples/genomic-loci meeting pre-

defined inclusion criteria were processed.

Reporting for specific materials, systems and methods

We require information from authors about some types of materials, experimental systems and methods used in many studies. Here, indicate whether each material, system or method listed is relevant to your study. If you are not sure if a list item applies to your research, read the appropriate section before selecting a response.

Materials & experimental systems

n/a	Involved in the study
☐	☒ Antibodies
☐	☒ Eukaryotic cell lines
☒	☐ Palaeontology and archaeology
☐	☒ Animals and other organisms
☒	☐ Clinical data
☒	☐ Dual use research of concern
☒	☐ Plants

Methods

n/a	Involved in the study
☐	☒ ChIP-seq
☒	☐ Flow cytometry
☒	☐ MRI-based neuroimaging

Antibodies

Antibodies used: CTCF antibody (rabbit polyclonal, Merck Millipore 07-729, lot 2517762).

Validation: From Merck Millipore : https://www.merckmillipore.com/GB/en/product/Anti-CTCF-Antibody,MM_NF-07-729
Routinely evaluated by Western Blot.

Western Blot Analysis:
A 1:1000–1:5000 dilution of this lot detected CTCF in HeLa nuclear extract. A previous lot detected CTCF in K562 nuclear extract (data not shown).
Application - Use Anti-CTCF Antibody (Rabbit Polyclonal Antibody) validated in ChIP, WB, ChIP-seq to detect CTCF also known as 11-zinc finger protein, CCCTC-binding factor.
The same antibody has also been used and validated in previous publications, for example https://doi.org/10.1186/s13059-018-1484-3

Eukaryotic cell lines

Policy information about cell lines and Sex and Gender in Research

Cell line source(s): The Hepa1-6 cell line was obtained from ATCC (accession: CRL-1830). The Hep-74.3a cell line was obtained from biohippo (accession BHC18001713).

Authentication: Genome sequencing confirmed expected mouse strains for both cell lines.

Mycoplasma contamination: Cell lines were newly acquired from sources for this study, tested and found negative for mycoplasma at source.

Commonly misidentified lines (See ICLAC register): None

Animals and other research organisms

Policy information about studies involving animals; ARRIVE guidelines recommended for reporting animal research, and Sex and Gender in Research

Laboratory animals: Mus musculus C3H/HeOuJ strain postpartum 15 days (P15), male.

Wild animals: This study did not involve wild animals.

Reporting on sex: As the study built on a large pre-existing dataset of tumours induced specifically in male mice (Aitken et al., Nature 2020), matched animals were use for the new sequence data reported in this study. Tissue samples for ATAC-seq and ChIP-seq were collected contemporaneously from the same colony as the original study.

Field-collected samples: This study did not involve field-collected samples.

Ethics oversight: Animal experimentation was carried out in accordance with the Animals (Scientific Procedures) Act 1986 (United Kingdom) and with the approval of the Cancer Research UK Cambridge Institute Animal Welfare and Ethical Review Body (AWERB).

Note that full information on the approval of the study protocol must also be provided in the manuscript.

Plants

Seed stocks	Not applicable
Novel plant genotypes	Not applicable
Authentication	Not applicable

ChIP-seq

Data deposition

☒ Confirm that both raw and final processed data have been deposited in a public database such as GEO.

☐ Confirm that you have deposited or provided access to graph files (e.g. BED files) for the called peaks.

Data access links<br>*May remain private before publication.*	ChIP-seq data submitted to Array Express, accession E-MTAB-11959. ttps://www.ebi.ac.uk/arrayexpress/experiments/E-MTAB-11959/<br>ATAC-seq data are available from Array Express at EMBL-EBI under accession E-MTAB-11780. https://www.ebi.ac.uk/arrayexpress/experiments/E-MTAB-11780/
Files in database submission	*Provide a list of all files available in the database submission.*
Genome browser session<br>(e.g. UCSC)	*Provide a link to an anonymized genome browser session for "Initial submission" and "Revised version" documents only, to enable peer review. Write "no longer applicable" for "Final submission" documents.*

Methodology

Replicates	5 biological replicates.
Sequencing depth	ChIP-seq was 150bp paired end reads. Semi-colon delimited fields for CTCF ChIP-seq libraries are:<br>library_identifier; total_read_count; uniquely_mapped_read_count<br>do17757; 40,870,808; 33,468,454<br>do17797; 51,230,864; 41,989,740<br>do17839; 22,059,191; 18,232,544<br>do18187 44,945,921 36,979,824<br>do18326 51,904,233 43,086,293
Antibodies	CTCF antibody (rabbit polyclonal, Merck Millipore 07-729, lot 2517762)
Peak calling parameters	To identify ChIP-seq positive regions, we trimmed the HiSeq sequencing reads to 50 bp and then aligned them using BWA (v.0.7.17) using default parameters. Uniquely mapping reads were selected for further analysis. Peaks were identified for each ChIP library and input control using MACS2 (v.2.1.2) callpeak with default parameters, and all peaks with a q-value >0.05 were included in downstream analyses. Input libraries were used to filter spurious peaks associated with a high input signal using the GreyListChIP R package (Brown 2021).
Data quality	Biologically-reproducible peaks were identified by merging ChIP-seq peaks defined as above (with an FDR q-value >0.05) from individual replicates and selecting those that overlapped ≥2 individual replicate peaks.
Software	MACS2 (v.2.1.2) callpeak with default parameters
